# Small for Gestational Age and Magnesium: Intrauterine magnesium deficiency may induce metabolic syndrome in later life

**DOI:** 10.3934/publichealth.2015.4.793

**Published:** 2015-12-04

**Authors:** Junji Takaya

**Affiliations:** Department of Pediatrics, Kawachi General Hospital, Higashi-Osaka, Osaka 578-0954, Japan

**Keywords:** DOHaD, fetal programming, metabolic syndrome, SGA

## Abstract

Magnesium deficiency during pregnancy as a result of insufficient or low intake of magnesium is common in developing and developed countries. Previous reports have shown that intracellular magnesium of cord blood platelets is lower among small for gestational age (SGA) groups than that of appropriate for gestational age (AGA) groups, suggesting that intrauterine magnesium deficiency may result in SGA. Additionally, the risk of adult-onset diseases such as insulin resistance syndrome is greater among children whose mothers were malnourished during pregnancy, and who consequently had a low birth weight. In a number of animal models, poor nutrition during pregnancy leads to offspring that exhibit pathophysiological changes similar to human diseases. The offspring of pregnant rats fed a magensium restricted diet have developed hypermethylation in the hepatic 11β-hydroxysteroid dehydrogenase-2 promoter. These findings indicate that maternal magnesium deficiencies during pregnancy influence regulation of non-imprinted genes by altering the epigenetic regulation of gene expression, thereby inducing different metabolic phenotypes. Magnesium deficiency during pregnancy may be responsible for not only maternal and fetal nutritional problems, but also lifelong consequences that affect the offspring throughout their life. Epidemiological, clinical, and basic research on the effects of magnesium deficiency now indicates underlying mechanisms, especially epigenetic processes.

## 1. Introduction

Maternal malnutrition is a well-known causal factor for intrauterine fetal growth retardation, both in humans and animals. Several studies have shown that small birth size and indices in poor fetal growth are associated with the development of metabolic syndrome in adulthood, such as insulin resistance, type 2 diabetes, hypertension, dyslipidemia and coronary heart disease [1]–[3]. Studies have indicated that exposure to adverse environmental influences during critical periods of development may cause persistent changes in organ structure and function and later lead to impaired glucose tolerance and type 2 diabetes, a phenomenon known as “programming” [2]–[4]. As birth size is mainly determined by non-genetic factors, these findings have led to the “developmental origins of health and disease (DOHaD)” hypothesis, whereby abnormal fetal growth as a result of fetal adaptation adverse intrauterine conditions may program lifelong physiological changes [5]–[8].

In spontaneous pregnancies smaller increases of thyroid stimulating hormone levels are related to higher magnesium (Mg) levels [9]. It has been reported that maternal intake of Mg is associated not only with fetal development, but also with the health of the newborn [10]. A daily intake of Mg during pregnancyis recommended as 1.40 mmol/day [11]. In India 44% of pregnant women consume less Mg in their diet [12]. Another study has demonstrated that pregnant mothers who drink water with a high Mg content lower their risk of having very low birth weight infants (less than 1,500 g of birth weight) [13].

The role of Mg as a cofactor for enzymes in carbohydrate metabolism also involves insulin activity [14]. Mg deficiency has been observed in cases of diabetes and vascular disease [15]–[18]. Lowere plasma Mg concentrations (less than 0.83mmol/L) was associated with insulin resistance in steady-state plasma insulin response to continuous glucose infusion [15]. The results from these experimental and epidemiological studies suggest that Mg levels and birth weight are key determinants of insulin resistance.

In this review, we propose that intrauterine Mg deficiency may later manifest as metabolic syndrome in adulthood, and discuss the association of Mg regulation with low birth weight and metabolic syndrome.

## 2. Magnesium in fetus and baby

Small for gestational age (SGA) babies are those who are smaller in size than normal by gestational age and are commonly defined as having a birth weight below the 10th percentile by gestational age [19]. Mineral concentrations of meconium in SGA newborns and those of appropriate for gestational age (AGA) newborns of similar gestational age were compared to determine whether differences may provide clues of possible nutritional deficits of SGA infants. The concentration of minerals in meconium is known to be indicative of minerals used by the fetus and minerals supplied to the fetus by the mother [20]. In the less than 35-week subgroups, the SGA group had lower meconium iron and manganese concentrations than those of the AGA group. Among more than 36-week newborns, SGA infants had a higher copper concentration than AGA infants when adjusted for birth-rate. No differences were observed in zinc, calcium (Ca), Mg, or phosphorus concentrations. These results may reflect either a greater use of minerals or a decreased maternal supply [20].

### 2.1. Placental transport of magnesium

The levels of total Ca, ionized Ca, and Mg are higher in fetal circulation than in maternal blood [21]. Copper and selenium share the same transport pathway in the placental membrane along a concentration gradient in the maternal-fetal direction, whereas active transport largely influences the transfer of Mg and iron [22]. In fact, evidence for the existence of an active transport mechanism for Mg in the placenta was suggested by using cultured trophoblast cells, i.e. a functional Na^+^/Mg^2+^ exchanger that functions to maintain low intracellular Mg [23]. The activity of this exchanger might be influenced by maternal plasma sodium concentration as experimental rats with acute maternal hyponatremia showed reduced maternal-fetal transfer of Mg via the placenta [24], while other pathways of Mg transport may also exist in the placenta. Recently, Yang H et al reported that magnesium/inorganic phosphorus channels expression is down-regulated in cases of preeclampsia and under hypoxia [25]. By the end of a normal pregnancy, the fetus is believed to have acquired approximately 28 g of calcium, 16 g of phosphorous, and 0.7 g of Mg, mostly during the third trimester, also the time when 80% of fetal accretion of Mg occurs [26]. Whereas mean levels of ionized Ca do not change during labor, the mean maternal serum levels of ionized Mg and total Mg fall at delivery, suggests the presence of homeostatic mechanisms in the fetus and placenta and indicating that free Mg in umbilical venous blood may enhance Mg transport to the fetus [27].

In mammals, the placenta is a highly developed organ which, along with nutrient exchange, has numerous other functions during the gestational period with maternal-fetal homeostasis dependant on a properly functioning placenta. Maternal Mg deficiency obviously affects the health of the fetus. However, some studies have not found a significant association between maternal trace elements and birth weight [28].

### 2.2. Placental vascular flow and magnesium

Ca and Mg are cofactors in the synthetic activity of a number of enzymes. A variety of hormones, cytokines and growth factors produced by fetal membranes and placenta can act locally on the myometrium [29]. It has been suggested that upregulation of multiple pathways involved in the production of nitric oxide (NO) is connected to the dilation of the uterine artery during pregnancy [30]. While the activity of constitutive NO synthase is dependent on Ca, the enzyme can also be inhibited by a reduction in the concentration of Mg [31]. In cases of preterm labor, the fetal membrane has a reduced permeability to Ca and Mg which could be an important factor in the activation of the myometrium in these cases [32].

**Figure 1.**
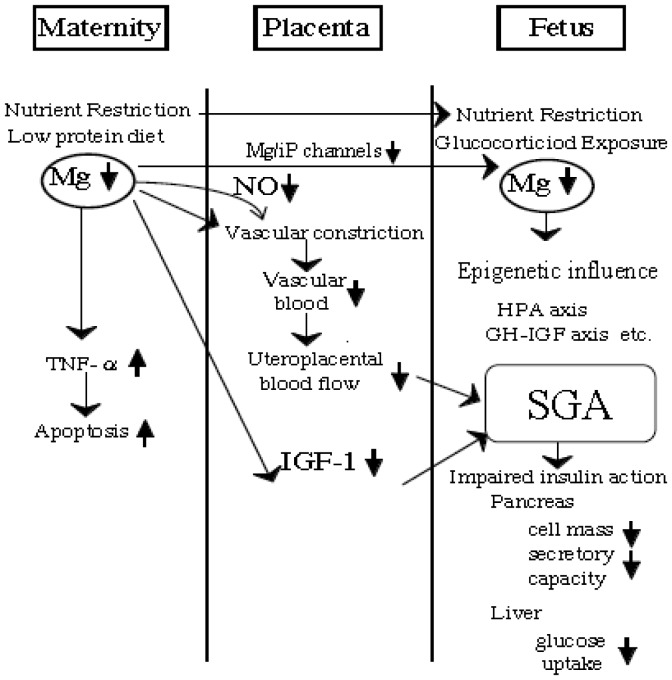
A placenta possesses an active transport mechanism for magnesium.

Mg along with Ca and NO has an immediate effect on placental vascular flow. Reduced placental vascular flow is at least, in part, responsible for placental insufficiency and SGA (Figure 1).

A variety of hormones, cytokines and growth factors produced by fetal membranes and placenta can act on the myometrium. The ability of the uterine artery to dilate may be related to nitric oxide. Several candidate processes have been proposed to explain gestational programming, i.e. epigenetic modification of gene. (adapted from Takaya J, Yamato F, Kaneko K. (2006) Possible relationship between low birth weight and magnesium status: from the standpoint of “fetal origin” hypothesis. Magnes. Res. 20, 63-9) IGF-1: insulin-like growth factor-1, iP: inorganic phosphorus, NO: Nitric oxide, Mg: magnesium, SGA: small for gestational age.

### 2.3. Intracellular Mg in cord blood platelets

Human platelets are often used in studying cellular cation metabolism in various diseases [33] because they are readily available and are thought to share a number of features with vascular smooth muscle cells. Additionally, insulin receptors in platelets have similar characteristics to those of other cells [34].

We and other investigators have proposed that insulin action could be mediated by intracellular Mg ([Mg^2+^]_i_) in platelets [35],[36]. In fact, Mg deficiency occurs in adult patients with diabetes mellitus and vascular diseases [16]–[18] and children with diabetes and obesity have been reported to have [Mg^2+^]_i_ deficiency [35].

Together, this evidence shows that decreased [Mg^2+^]_i_ might underlie the initial pathophysiologic events leading to insulin resistance and further tested whether the origin of [Mg^2+^]_i_ deficiency may start from fetal life in SGA caused by genetic factors or intrauterine environment.

***(A) Intracellular Mg and small for gestational age***

**Figure 2.**
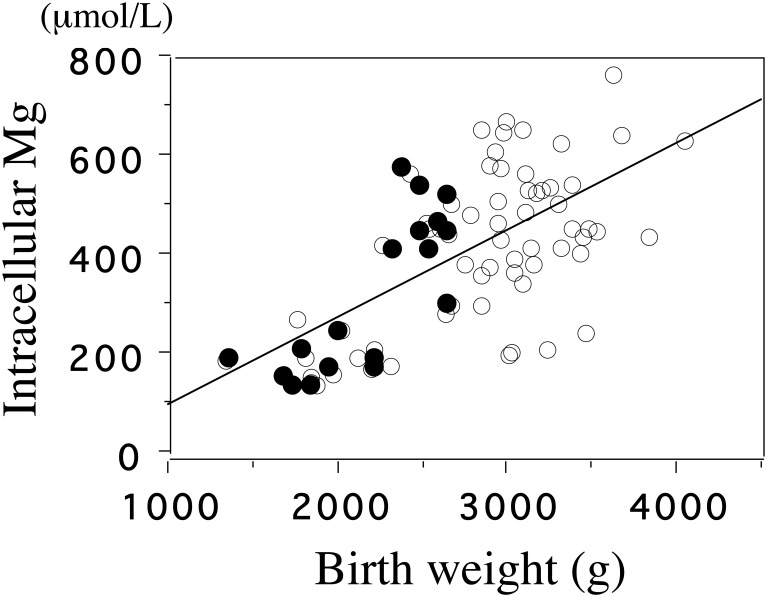
The correlation between intracellular Mg and birth weight.

**Figure 3.**
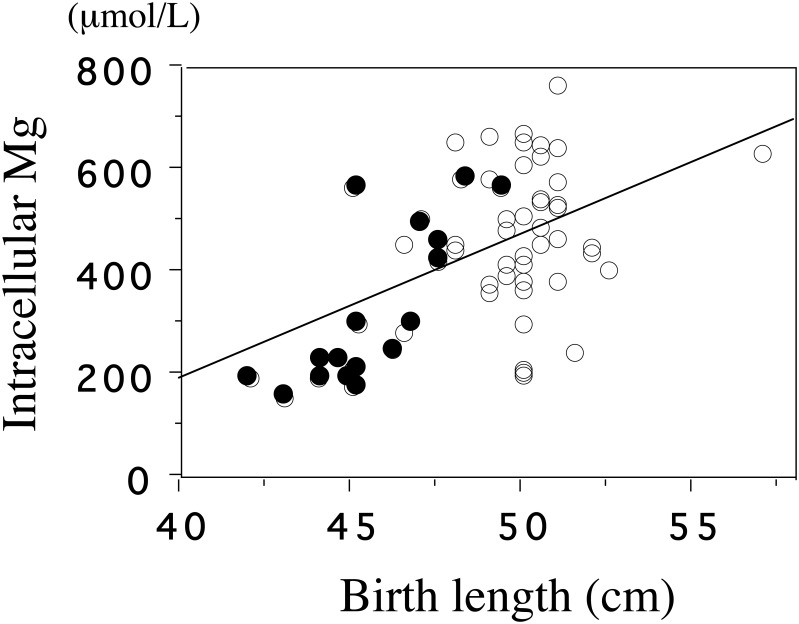
The correlation between intracellular Mg and birth length.

By using a fluorescent probe, mag-fura-2, we examined [Mg^2+^]_i_ of platelets in the cord blood of infants with SGA and with AGA [36]. Mean [Mg^2+^]_i_, but not plasma Mg, was lower in the SGA than in the AGA group (284 ± 33 μmol/L vs 468 ± 132 μmol/L, *p* < 0.001). [Mg^2+^]_I_ was significantly correlated with the birth weight (*p* < 0.001) and birth length (*p* < 0.001) (Figure 2,3).

As [Mg^2+^]_i_ plays a promotive role in fetal growth, low [Mg^2+^]_i_ may partly be responsible for SGA. In regard to fetal life, it has been postulated that nutritional and environmental factors during pregnancy, as well as hormonal factors such as insulin and IGF-1 [37] play important roles in addition to genetic predisposition.

The basal level of intracellular Mg^2+^ of cord blood platelets is significantly correlated with birth weight (*p* < 0.001, *r* = 0.61). &xcirc;, appropriate for gestational age (AGA); **•**, small for gestational age (SGA) (adapted from Reference 36)

The basal level of intracellular Mg^2+^ of cord blood platelets is significantly correlated with birth length (*p* < 0.001, *r* = 0.48). &xcirc;, appropriate for gestational age (AGA); **•**, small for gestational age (SGA). (adapted from Reference 39)

***(B) Correlation of [Mg^2+^]_i_ and insulin resistance***

**Figure 4.**
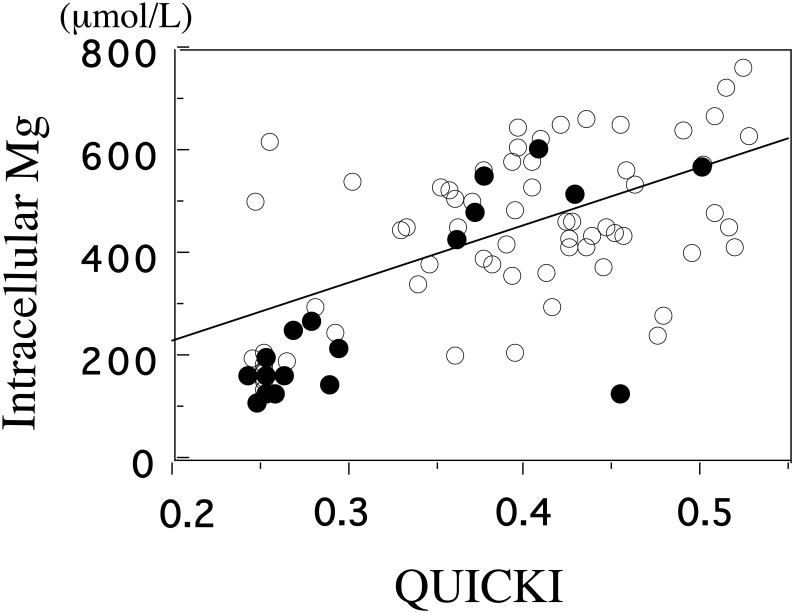
The correlation between intracellular Mg and QUICKI.

Adiponectin and IGF-1 were lower in SGA than in AGA groups, while plasminogen activator inhibitor (PAI)-1 and ghrelin were higher in SGA groups (Table 1). Quantitative insulin sensitivity check index (QUICKI) has an excellent linear correlation with the glucose clamp index of insulin sensitivity [38]. Homeostasis Model Assessment-Insulin Resistance (HOMA-IR) and QUICKI are the most widely used indices for assessing insulin sensitivity. QUICKI was lower in SGA than in AGA groups (Table 1). Birth weight was correlated with cord plasma IGF-1 (*p* < 0.001), adiponectin (*p* < 0.001) and leptin (*p* < 0.005). [Mg^2+^]i, and adiponectin were correlated with QUICKI in all subjects (Fig 4 )[39]. These results show that SGA individuals have the tendencies toward insulin resistance. [Mg^2+^]_i_ was significantly associated with adiponectin (*r* = 0.246, *p* = 0.04), IGF-1(*r* = 0.272, *p* < 0.03), QUICKI (*r* = 0.592, *p* < 0.0001)(Figure 4). From these findings, [Mg^2+^]_i_ as well as leptin and IGF-1 can be used to indicate the extent of fetal growth. Lower [Mg^2+^]_i_ may be involved in the underlying processes that result in insulin resistance.

In summary low [Mg^2+^]_i_, may influence the prenatal programming of insulin resistance, and may have lifelong effects on metabolic regulation characterized by insulin resistance.

The basal level of intracellular Mg^2+^ of cord blood platelets is significantly correlated with QUICKI (*p* < 0.001, *r* = 0.59). &xcirc;, appropriate for gestational age (AGA); **•**, small for gestational age(SGA). (adapted from Reference 39)

**Table 1:** A comparison between the metabolic hormones of SGA and AGA infants

	SGA (n = 20)	AGA (n = 45)
[Mg^2+^]i (μmol/L)	284 ± 33***	468 ± 132
Plasma Mg(mmol/L)	0.60 ± 0.03	0.61 ± 0.02
Adiponectin(μg/mL)	11.4 ± 1.8**	17.1 ± 1.0
IGF-1(ng/mL)	14.3 ± 2.1**	30.3 ± 2.2
Leptin(pg/mL)	845 ± 215	1,260 ± 137
Ghrelin(fmol/mL)	76.8 ± 11.0*	53.7 ± 5.1
PAI-1(ng/mL)	13.20 ± 2.52*	7.97 ± 0.94
QUICKI	0.35 ± 0.02***	0.41 ± 0.01

SGA: small for gestational age, AGA: appropriate for gestational age, [Mg^2+^]i: intracellular magnesium, PAI-1: plasminogen activator inhibitor-1, QUICKI: Quantitative insulin sensitivity check index. *p < 0.05, **p < 0.005, ***p < 0.0001 (adapted from Reference 39)

### 2.4. Fetal programming

The fetal origin hypothesis by Barker et al. [3],[6],[7] states that fetal undernutrition in middle to late gestation leads to disproportionate fetal growth programs and later metabolic diseases. Fetal programming is a phenomenon in which alterations in fetal growth and development in response to the prenatal environment have long-term or permanent effects. This theory was further established in the category of DOHaD [8]. The mechanisms connected with fetal programming include the direct effects on cell number, altered stem cell function, and the resetting of regulatory hormonal axes; hypothalamic-pituitary-adrenal axis [40],[41] and growth hormone insulin-like growth factor axis [42]. One of the underlying mechanisms for the postulated early-life programming is epigenetics. Altered epigenetic regulation of genes in phenotype induction could possibly give rise to interventions that modify long-term disease risk associated with unbalanced nutrition in early life [43].

Although low birth weight and poor prenatal nutrition are strongly associated with metabolic syndrome in later life, postnatal catch-up growth was recently considered to also be a pivotal element associated with the development of various pathological conditions [44]. The concept of a sensitive or crucial period that whereby the fetus is susceptible to long-term changes in development and adverse outcomes later in life is an intriguing one and should be the focus of future studies.

(1) Fetal programming

**Figure 5.**
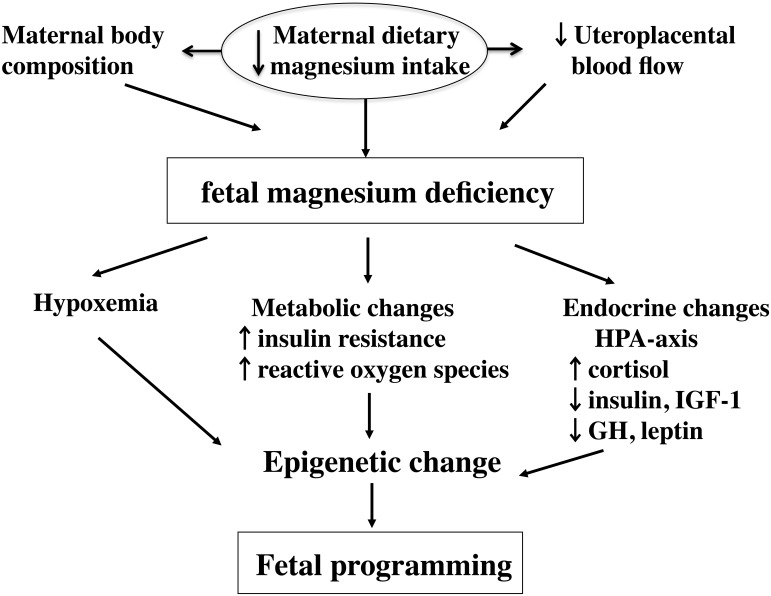
Fetal programming

Fetal programming is the phenomenon whereby alteration in fetal growth and development in response to the prenatal environment has long-term or permanent effects. The mechanisms involved are believed to have a direct effect on cell number, altered stem cell function and resetting of regulatory hormonal axes (Figure 5).

There are several candidates that may be involved in gestational programming: [i] a potential role for the hypothalamic-pituitary-adrenal axis has been suggested, as the mediators of the fetal response to nutrient stress, i.e. maternal low protein diet, were profoundly suppressed [45]; [ii] fetal programming of the growth hormone insulin-like growth factor axis also has been proposed to serve as a link between fetal growth and adult-onset disease [42].

(2) Thrifty phenotype hypothesis

The “thrifty phenotype hypothesis”, which postulates that fetal programming for adaptation to an adverse intrauterine environment results in lower insulin sensitivity in utero, is one of the hypotheses to explain the association between low birth weight and insulin resistance in later life [46].

Decreased [Mg^2+^]i in infants with SGA can be the initial pathophysiologic events of fetal programming. A recent animal study supported our data demonstrating that the maternal Mg restriction irreversibly increases body fat and induces insulin resistance in pups by 6 months of age [47].

(3) Epigenetic modification of gene expression

It is intriguing in clinical practice that the intrauterine environment can program adult disease susceptibility by altering the epigenetic state of the fetal genome, hence affecting the phenotype without changing the DNA sequence [48]. The changes in the intrauterine environment may ultimately lead to altered gene expression via alterations in DNA methylation and other epigenetic mechanisms, resulting in an increased susceptibility to chronic disease in adulthood [49].

Biological methylation reaction is dependent on dietary methyl donors and on cofactors. Mg acts as a cofactor for the binding of protein to its specific site in DNA by inducing conformational changes in the protein [50]. Mg also changes the conformation to a more helical structure which could provide specific geometrical constraints complementary to those of DNA helix.

### 2.5. Animal models

Quantitative real-time PCR-based methylation analysis of three glucocorticoid related genes was performed from liver samples of offspring. In a rat model we found that a Mg-deficient diet in pregnant rats induces hypermethylation of specific CpG dinucleotides in the hepatic 11β-hydroxysteroid dehydrogenase-2 (Hsd11b2) promoter of infantile offspring [51]. These results show that nutrient constraint before birth induces persistent CpG-specific changes to the epigenetic regulation of the Hsd11b2 promoter and that these changes are associated with altered mRNA expression. Venu et al. reported that fasting insulin and HOMA-IR underwent an increase in the offspring of Mg restricted dams at 6 months of age [47]. Maternal and postnatal Mg status is important in the long-term programming of body adiposity and insulin secretion in rat offspring [52].

In addition to the classical mechanisms of gene deletion or inactivation by point mutations, some genes can be functionally inactivated by methylation of cytosine residues in the promoter region without any alteration to their primary sequences. Epigenetic regulation of gene transcription provides a strong candidate mechanism for fetal programming. Inactivation of genes is an important event contributing to the development of metabolic diseases. Changes in Hsd11b2 probably contribute to marked increases in glucocorticoid hormone action in tissues and thereby potentiate the induction of insulin resistance in adult life.

Mg deficiency promotes apoptosis in cardiovascular cells, oxidative stress, and DNA damage [53]. Recently, Shah et al reported that Mg deficiency results in downregulation of telomerase and upregulation of transcription factores in rat cardiac and aortic smooth muscle cells [54].

## 3. Conclusion

Birth weight is only a crude index of early growth and does not indicate the success of a fetus in achieving its growth potential. [Mg^2+^]_i_ may be a marker of early growth restriction, which may be of future diagnostic use as an early predictor of adult diseases. Low [Mg^2+^]_i_, which may represent the prenatal programming of insulin resistance, has lifelong effects on metabolic regulation. A biological interpretation of the association between birth size and risk of insulin-resistant diseases should emphasize the possible underlying roles of [Mg^2+^]_i_.
